# Microglia-derived exosomes selective sorted by YB-1 alleviate nerve damage and cognitive outcome in Alzheimer’s disease

**DOI:** 10.1186/s12967-024-05256-x

**Published:** 2024-05-16

**Authors:** Hong Wei, Zhuzhi Zhu, Yuhao Xu, Li Lin, Qi Chen, Yueqin Liu, Yuefeng Li, Xiaolan Zhu

**Affiliations:** 1 Reproductive Center, The Fourth Affiliated Hospital of Jiangsu University, 20 Zhengdong Road, Zhenjiang, 212001 Jiangsu P. R. China; 2 Department of Neurology, The Fourth Affiliated Hospital of Jiangsu University, Zhenjiang, 212001 Jiangsu China; 3 Central Laboratory of the Fourth Affiliated Hospital of Jiangsu University, Zhenjiang, 212001 Jiangsu China; 4 Department of Orthopedics, Nanjing Lishui People’s Hospital, ZhongDa Hospital Lishui Branch, Southeast University, Nanjing, 212001 Jiangsu China; 5 Medical School, Jiangsu University, 301 Xuefu Road, Zhenjiang, 212001 Jiangsu P. R. China

**Keywords:** Microglia, miR-223, YB-1, EXO sorting, AD

## Abstract

**Background:**

Neuroinflammation is a characteristic pathological change of Alzheimer’s Diseases (AD). Microglia have been reported to participate in inflammatory responses within the central nervous system. However, the mechanism of microglia released exosome (EXO) contribute to communication within AD microenvironment remains obscure.

**Methods:**

The interaction between microglia and AD was investigated in vitro and in vivo. RNA-binding protein immunoprecipitation (RIP) was used to investigate the mechanisms of miR-223 and YB-1. The association between microglia derived exosomal YB-1/miR-223 axis and nerve cell damage were assessed using Western blot, immunofluorescence, RT-PCR, ELISA and wound healing assay.

**Results:**

Here, we reported AD model was responsible for the M1-like (pro-inflammatory) polarization of microglia which in turn induced nerve cell damage. While M2-like (anti-inflammatory) microglia could release miR-223-enriched EXO which reduced neuroinflammation and ameliorated nerve damage in AD model in *vivo* and in *vitro*. Moreover, YB-1 directly interacted with miR-223 both in cell and EXO, and participated in microglia exosomal miR-223 loading.

**Conclusion:**

These results indicate that anti-inflammatory microglia-mediated neuroprotection form inflammatory damage involves exporting miR-223 via EXO sorted by YB-1. Consequently, YB-1-mediated microglia exosomal sorting of miR-223 improved the nerve cell damage repair, representing a promising therapeutic target for AD.

**Supplementary Information:**

The online version contains supplementary material available at 10.1186/s12967-024-05256-x.

## Introduction

Accumulating documents identified that neuroinflammation is a central mechanism in Alzheimer’s disease (AD) [1]. The microglia are the predominant immune cell in central nervous system, and microglia dysfunction is reported to be contribution of the onset and/or progression in AD [2]. New evidence indicated that aggregated Aβ could activate microglia and impair the phagocytotic ability [3]. On the other hand, activation of microglia towards an anti-inflammatory state had therapeutic potential in AD [4]. In sum, microglia could have either beneficial or detrimental effects during the onset and progression of AD, depending on their polarization. Lately, immunotherapy has been considered the most visible strategy for the treatment of AD [5], and modifying the activated state is clearly a trend for future studies.

Exosome (EXO) is 30–150 nm nanovesicles of endosomal origin, enriched in nucleic acids, lipids, and proteins that could mediate intercellular communication in normal physiology and pathology [6, 7]. MiRNAs which are commonly enriched in EXO, are considered as potent modulators of cellular processes, such as neuroinflammation, differentiation and migration [8]. In our previous study we found a dysregulation of neuronal-derived exosomal miR-223 was associated with nerve damage in AD [9]. In addition, recent advances in therapy of M2 microglia-derived EXO have also enabled absorption of exosomal miRNAs to modulate inflammatory responses [10]. Indeed, our previous studies found that miR-223 movement from mesenchymal stem cell (MSC) to AD cell model through EXO could protect against nerve injury. However, the study of exosomal miR-223 in AD has just begun, and whether microglia-derived EXO-packaged miR-223 could attenuate nerve damage remains unknown.

Cellular stress could induce unique changes in the abundance of these exosomal molecules [11], indicating that cargo sorting into EXO is not a passive process and there must be a specific mechanism for loading miRNAs [12]. In fact, several pathways and molecules, including RNA binding proteins (RBPs), have been described that impact exosomal miRNAs loading [13]. RBPs are crucial regulators of the post-transcriptional processing and transport of RNA molecules, and their directly interact with the components of multivesicular body (MVB), raising the possibility that RBPs may be related to exosomal miRNA loading [14]. Therefore, RBPs may play a role in determining which RNAs or miRNAs are selected for sorting in EXO. Fengxia Lin et al. found that miR-133 was specially sorted into EXO via YB-1 [15]. Matthew J Shurtleff et al. found that YB-1 was necessary for packaging of miR-223 and it played an important role in the secretion of this miRNA into EXO in a cell-free reaction in 293T cell [14, 16]. Therefore, this study focused on the content and sorting mechanism of miR-223 in microglia EXO and the effect on the nervous system.

In this study, we sought to elucidate the role of microglia in AD immunotherapy, and to determine the sorting mechanism of miR-223 in microglia-derived EXO. Further discussing the application of exosomal miR-223 on AD treatment aimed to offer a comprehensive understanding of functionalizing microglia for AD immunotherapy.

## Materials and methods

### Reagents and antibodies

The amyloid β protein fragment 1–40 (Aβ_1−40_), GW4869, PKH67, LPS and IFN-γ were purchased from Sigma (California, USA). Lipofectamine 2000 were purchased from Invitrogen (CA, USA). The antibodies GAPDH, YB-1, PTEN, NeuN and Iba1 were purchased from Abcam (Cambridge, MA), and the antibodies β3 tubulin, CD206, CD11b, Arg-1, iNOS were purchased from Cell Signaling Technology (Boston, Massachusetts). The ELISA kits were purchased from Blue gene (Shanghai, China). The YB-1 plasmids, miR-223 antagomir and miR-223 agomir were purchased from Genechem (Shanghai, China). The RiboCluster Profiler RIP-Assay Kit was purchased from MBL Medical & Biological Laboratories Co (LTD, Japan).

### Cell culture and co-culture system

Cultured human neuroblastoma SH-SY5Y cell was obtained from Chinese Academy of Sciences. When grew to 70%, cells were treated with Aβ_1−40_ at a final concentration of 5 µmol/L for AD cell model.

BV2 cell was purchased from American Type Culture Collection (ATCC). For microglia generation and differentiation, BV2 was first treated with 1 µg/mL LPS for 6 h (M0). The M0 was stimulated with 20 ng/mL IL-4 to obtain M2-like microglia and with 100 ng/mL LPS and 10 ng/mL IFN-γ to obtain M1-like microglia. The polarization of microglia was confirmed by detecting specific protein signatures with iNOS, CD206, CD11b, Arg-1 and IL-10.

All animal procedures were performed in accordance with the National Institutes of Health Guide for the Care and Use of Laboratory Animals and were approved by the Animal Ethics Committee of the Jiangsu University. The SD rats used in this study were maintained from the Shanghai SLAC Laboratory Animal Company. Primary neuron (PN) were prepared according to previously published protocol [17]. Briefly, a pregnant female rat was anesthetized to death, and the embryos were promptly separated via cesarean section and placed in the cold balanced salt solution on ice. The cerebral cortex was carefully isolated and digested with a EDTA for 5 min. The precipitation was filtered with a sieve with a diameter of 40 μm, then centrifuge. The obtained cell suspension was added into 10% media. The cells could be used for experiment after 6 days.

Primary microglia (PM) were obtained from neonatal C57 mice as described previously [18]. Briefly, the brain tissues were dissected and digested, followed by mechanical shearing. After centrifugation, cells were filtered through a 70-um filter and cultivated in DMEM medium. Then incubated for 14 days, the microglial cells were separated from the underlying astrocytic monolayer.

In addition, microglia were co-cultured with AD cell model in a Transwell-6 system with a 0.4 μm porous membrane to allow EXO transfer and cell contact. Suppression of EXO release was performed by incubation with the N-SMase inhibitor GW4869 (20µM) for 8 h in microglia.

### Preparation and isolation of EXO

After inducing the phenotypes, the medium of BV2 cells was replaced with EV-free medium for 48 h. Then, the EXO pellets were collected from the supernatant of M2-like microglia. The EXO isolated from the supernatant of about 1 × 10^7^ cells (two 25cm^2^ petri dish), was used for one injection. The total volume of EXO for per mouse was approximately 2*10^10^.

The studies involving human participants were reviewed and approved by the Institutional Review Board of the Fourth Affiliated Hospital of Jiangsu University with informed consent from the patients. The serum from 20 first-visit AD patients and 20 age-matched healthy controls were collected for subsequent experiments.

EXO was isolated from serum or cell as previously described [9]. The isolation method comprised an additional centrifugation step to remove small cell debris followed by ultracentrifugation at 100,000×*g* for 1 h to generate an EXO pellet. The EXO from microglia was labeled with PKH67 or DiR for tracking EXO according to the protocol [19].

### miRNA expression profiling

Total RNA was extracted from cell and serum using Trizol reagent, and a FastQuant RT Kit (with gDNase) was used to generate cDNA from mRNA. Then, SYBR Premix was used for subsequent quantitative real-time PCR amplification following the manufacturer’s instructions. U6 was used as the internal control and the miRNA expression levels were analyzed by 2^−ΔΔCt^ method.

### Western blot analysis

Total protein was isolated with RIPA lysis buffer. For clinical validation, serum EXO from three individuals were mixed as one sample for Western Blot. Approximately 10 µg of protein was separated in 12% gels by sodium dodecyl sulfate-polyacrylamide gel electrophoresis, and then transferred to a PVDF membrane. Then membrane was blocked with 5% bovine serum albumin for 2 h and immunoblotted with antibodies. Chemiluminescence was detected with the ChemiDoc MP imager.

### Immunofluorescence

The cell was fixed with 4% paraformaldehyde for 15 min and permeabilized with 0.5% Triton X-100 which stopped by 5% bovine serum albumin. Then cell was immunostained using the corresponding antibody overnight and further labeled with the secondary antibody. The cell was fixed and stained with DAPI. The cell was detected by a Leica TCS sp5 II laser scanning confocal microscope.

### Cytokine measurement and wound healing assay

The concentration of IL-6, IL-1β, TNF-α and CRP were detected with ELISA kits in accordance with the manufacturer’s instructions. The absorbance values were measured at 450 nm and the standard curve was plotted to calculate the contents of IL-6, IL-1β, TNF-α and CRP.

The cell was seeded at a density of 1.2 × 10^4^ cell/well in a 6-well plate. The cell monolayer was scratched with a 200 µl pipette tip and observed by microscope.

### RNA immunoprecipitation assay

The M2-like microglia cell or EXO were performed using the Magna RIP RNA-binding protein immunoprecipitation kit and the YB-1 antibody following the manufacturer’s protocol. Co-precipitated RNAs were subjected to RT-qPCR analysis.

### In vivo behavior assays

At 5–14 days following the IV EXO treatment, cognitive tests including Morris water maze (MWM), novel object recognition (NOR), Y maze tests and open field test (OFT) were performed.

The MWM test evaluates spatial learning and memory. The mice were first subjected to 4 training trials per day for 7 consecutive days. At the end of the last trial, the durations that mice spent in the target quadrant and the crossings through the original location of the platform were recorded.

The NOR test evaluates recognition memory. During the training session, mice were exposed to the object for 10 min. For the test session, mice were put back to the familiar object for 5 min and new object for 5 min. The discrimination inde (NOR-DI) was computed as DI = (novel object exploration time - familiar object exploration time/total exploration time) *100. The discrimination ratio (NOR-DR) was computed as DR = (novel object exploration time/total exploration time) *100.

The Y maze test evaluates short-term memory. Mice were initially placed in the middle of maze, then freely moving 5 min. The alternation triplet (%) were used to define consecutive triplets of different arm choices.

The OFT evaluates locomotor activity and exploratory behavior. The area of the black square open field 1m^2^, and the bottom of the field was divided into 25 squares. The time spent in the center of the arena and total locomotion were measured.

### IVIS in vivo

To assess trafficking of EXO from M2-like microglia, DiR-labeled EXO was administered at five times the standard dose (5 × 10^6^ DiR-labeled EXO) and APP/PS1 mice were analyzed with an IVIS spectrum imager.

### Tunel assay

The brain slices fixed by 4% paraformaldehyde and were treated with 0.5% Triton X-100. Tunel reagent was added to slide, and brain slices were cultured in a dark environment. The fluorescent signals were detected with a fluorescence microscope.

### PET-CT imaging

Micro-PET-CT (Siemens) was used for dynamic body scan for mice. The mice were anesthetized and injected with [18 F] DPA-714 (0.2 ml/100 g). The scanning parameters were layer thickness 1 mm, matrix 128 × 128, current 500µA, voltage 80 kV and acquisition energy window 350-650 kV. The data was analyzed by Inveon Research Workplace (IRW 3.0) software and region of interest (ROI) analysis.

### Statistical analysis

Data are expressed as the mean ± SEM. Statistical analysis was performed using SPSS Statistics or GraphPad Prism. Significance was determined using Student’s t-test, one-way ANOVA or two-way ANOVA, as appropriate. The *p* value of less than 0.05 was statistically significant.

## Results

### AD model promotes M1-like (pro-inflammatory) microglia activation

**Fig. 1:**
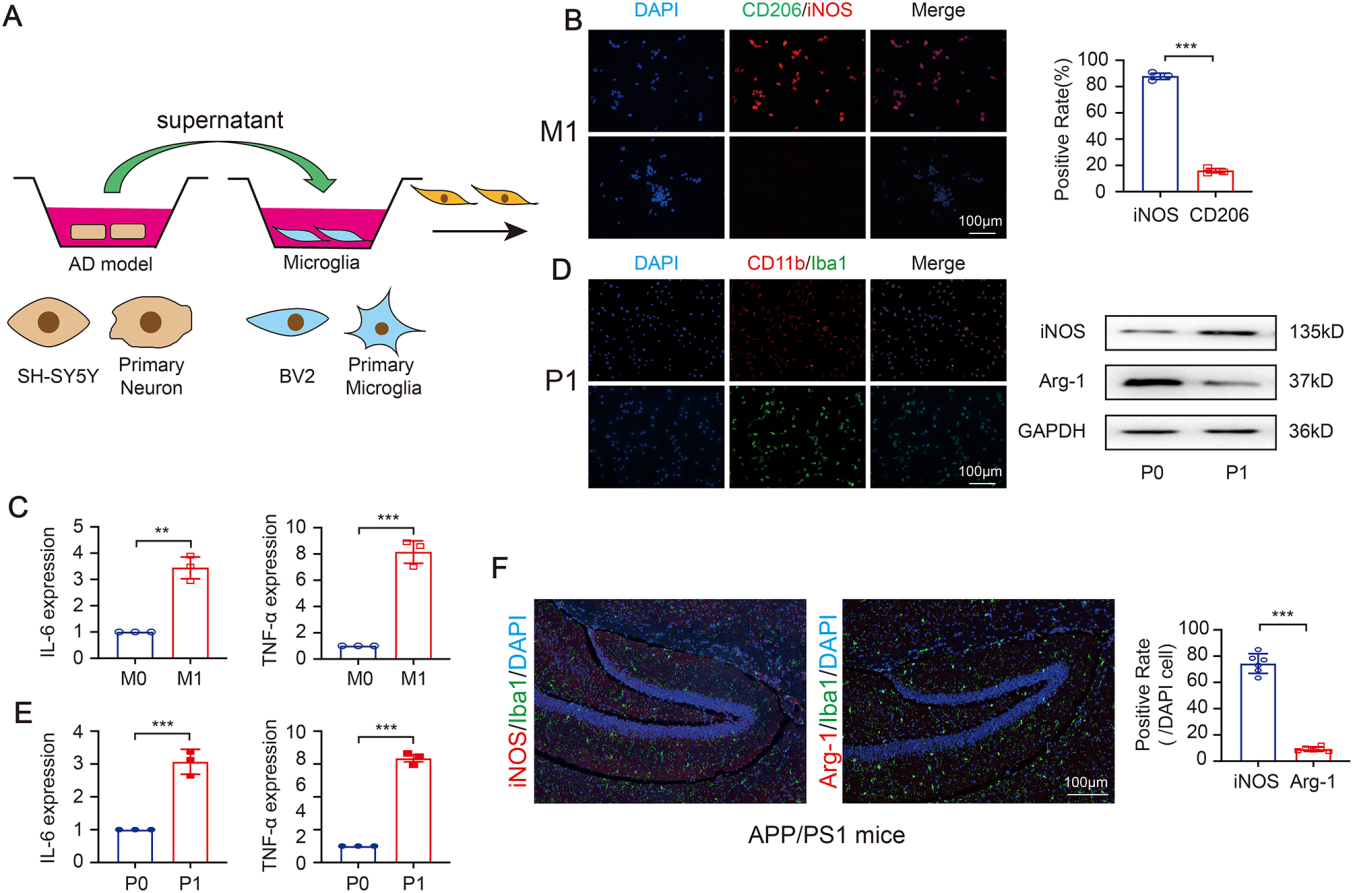
AD model promotes M1-like (pro-inflammatory) microglia activation. (**A**) Schematic diagram of the contact coculture system. (**B**) The representative immunofluorescence images of microglia makers CD206 and iNOS. (**C**) The express of IL-6 and TNF-α in treated BV2 (*N* = 3 independent experiments). (**D**) The representative immunofluorescence images of primary microglia (PM) makers CD11b and Iba1, and the expression of iNOS and Arg-1. (**E**) The express of IL-6 and TNF-α in treated PM (*N* = 3 independent experiments). (**F**) The polarized microglia in the hippocampus of brain tissue in APP/PS1 mice (*N* = 6 independent animals). Error bars represent means ± SEM. **P < 0.01, ***P < 0.001 vs. control

We first investigated the effect of AD model on microglia activation. The AD cell model was established as previously [19]. After differentiation, round and floating microglia cell became adherent flattened cell (Fig S1A). To determine whether AD cell model influence microglia, the supernatant from AD cell model were added into microglia for coculture (Fig. 1A). Not surprisingly, the expression of iNOS (iNOS^+^/CD206^−^) were observed in BV2 cells in the co-culture system (Fig. 1B), and treated BV2 cells showed an upregulated gene expression of IL-6 and TNF-α (Fig. 1C). Also, the supernatant from AD cell model established with primary neuron (PN) (a kind of cell could mimic the function of mouse brain to a certain extent [20]) were added into primary microglia (PM). The results showed that the pro-inflammatory microglia markers (CD11b^+^/Iba1^+^) were enhanced (Fig. 1D), and the cytokines (IL-6 and TNF-α) were upregulated (Fig. 1E). To further confirm the effect in *vivo*, the transgenic APP/PS1 mice, a recognized AD mouse model that could produce Aβ [21], was analyzed. And the pro-inflammatory microglia markers (iNOS^+^/Arg-1^−^) was also observed in hippocampus in APP/PS1 mice (Fig. 1F). The above results show that AD model could be responsible for the pro-inflammatory microglia.

### Pro-inflammatory microglia strengthen the progress of AD

**Fig. 2:**
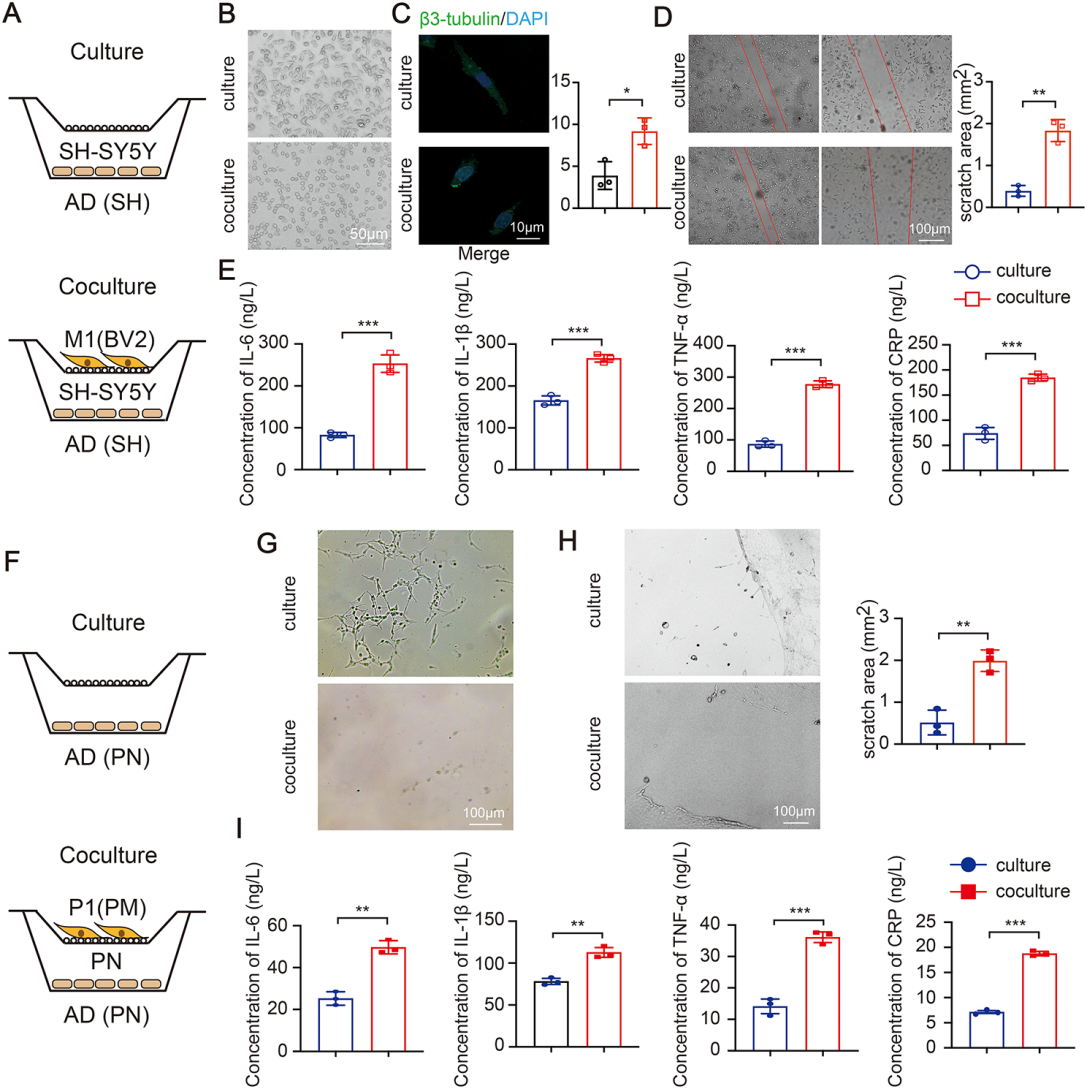
Pro-inflammatory microglia strengthen the progress of AD. (**A**) Schematic diagram of the culture or coculture system. Representative images of morphological characteristics (**B**), representative confocal images of β3-tubulin co-staining (**C**), wound healing assay (**D**) and four inflammatory cytokines (**E**) in AD cell model of SH-SY5Y cell. (**F**) Schematic diagram of the culture or coculture system between PN and PM. Morphological characteristics (**G**), wound healing assay (**H**) and four inflammatory cytokines (**I**) in AD cell model of PN. (*N* = 3 independent experiments). Error bars represent means ± SEM. **P < 0.01, ***P < 0.001 vs. control

Neuroinflammation, specifically microglia activation, has been reported as hall-markers of brain injury [22]. To this end, we employed a coculture system for pro-inflammatory microglia and AD cell model of SH-SY5Y cells (Fig. 2A). When cocultured, AD cell model showed reduced number of cell and shortened synapse (Fig. 2B). The changes of synaptic growth (Fig. 2C) and ultimate wound healing (Fig. 2D) showed a same tendency. Meanwhile, we found that AD cell model cocultured with pro-inflammatory microglia significantly enhanced the induction IL-6, IL-1β, TNF-α and CRP (Fig. 2E). The same trend was observed in the coculture system of PN and PM (Fig. 2F and I). Those observations emphasizes that pro-inflammatory microglia could promote nerve damage of AD cell model.

### M2-like (anti-inflammatory) microglia could reverse nerve damage

**Fig. 3:**
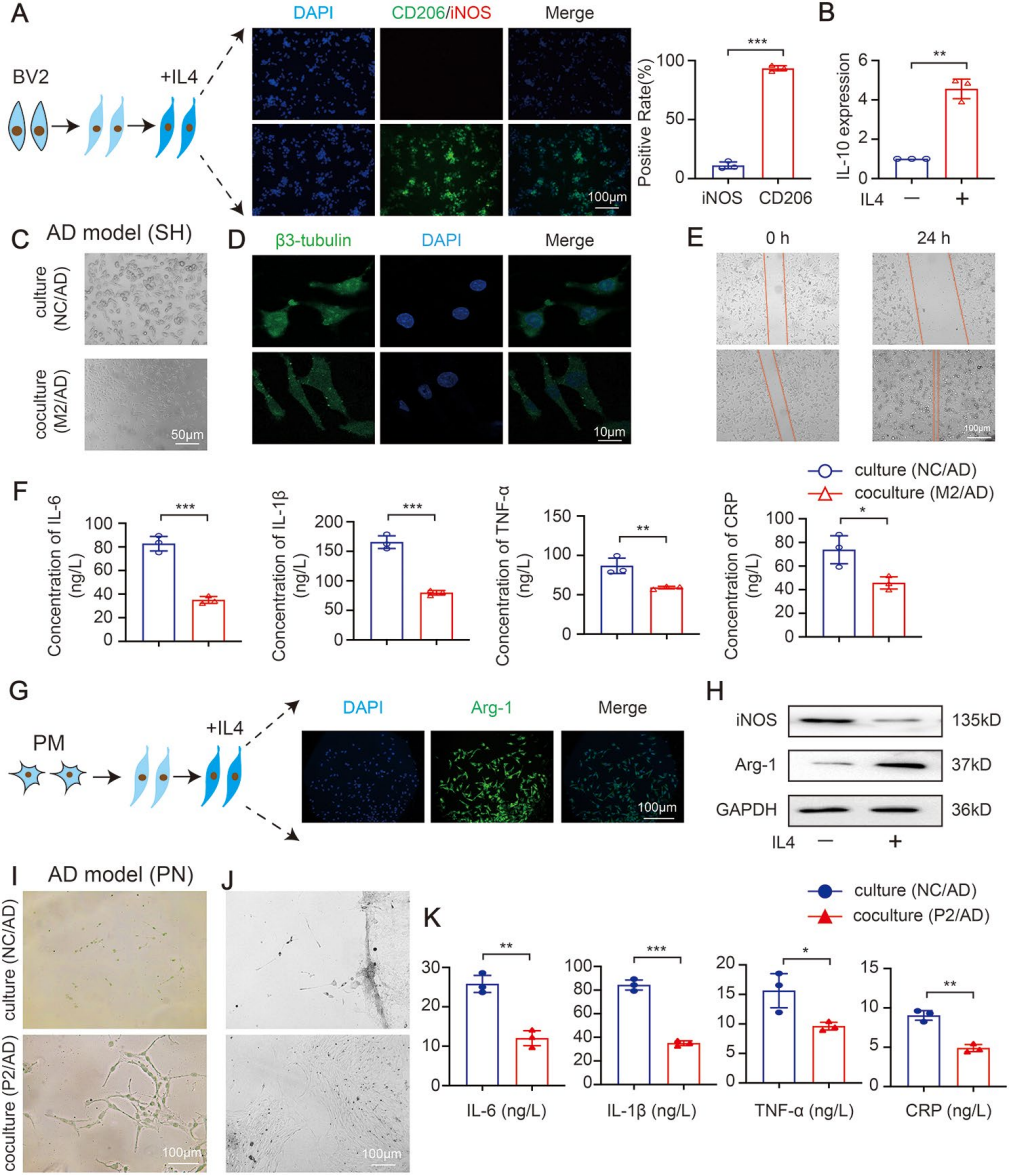
M2-like anti-inflammatory microglia could reverse nerve damage. (**A**) Representative immunofluorescence images of microglia markers in BV2. (**B**) The express of IL-10. Morphological characteristics (**C**), β3-tubulin immunofluorescence co-staining (**D**), wound healing assay (**E**) and four inflammatory cytokines (**F**) in AD cell model of SH-SY5Y cell. (**G**) Representative immunofluorescence images of microglia markers in PM. (**H**) The expression of iNOS and Arg-1. Morphological characteristics (**I**), wound healing assay (**J**) and four inflammatory cytokines (**K**) in AD cell model of PN. (*N* = 3 independent experiments). Error bars represent means ± SEM. *P < 0.05, **P < 0.01, ***P < 0.001 vs. control

Since pro-inflammatory microglia hold an effect on promoting the AD progression, in contrast, anti-inflammatory microglia may attenuate nerve injury. As such, microglia were induced to verify the above conjecture. In contrast to pro-inflammatory microglia, the generated anti-inflammatory microglia positively expressed CD206 but negatively expressed iNOS (iNOS^−^/CD206^+^) (Fig. 3A). And the express of IL-10 in anti-inflammatory microglia was increased (Fig. 3B). Next, morphology and function were assessed. As expected, AD cell model in non-contact cocultured with anti-inflammatory microglia showed an improved cell morphology (Fig. 3C), increased synapses (Fig. 3D) and decreased scratch area (Fig. 3E). The levels of inflammatory cytokines were significantly decreased in coculture system (Fig. 3F). Moreover, the induced anti-inflammatory microglia were cocultured with PN. In PM from the fetal rat brain, the generated anti-inflammatory microglia positively expressed Arg-1 and negatively expressed iNOS (Fig. 3G-H). And the anti-inflammatory microglia similarly improved cell morphology (Fig. 3I), decreased scratch area (Fig. 3J), and downregulated inflammation (Fig. 3K). These results provided the evidence that anti-inflammatory microglia could reverse nerve damage.

### Anti-inflammatory microglia derived exosomal miR-223 to improve AD cell model

**Fig. 4:**
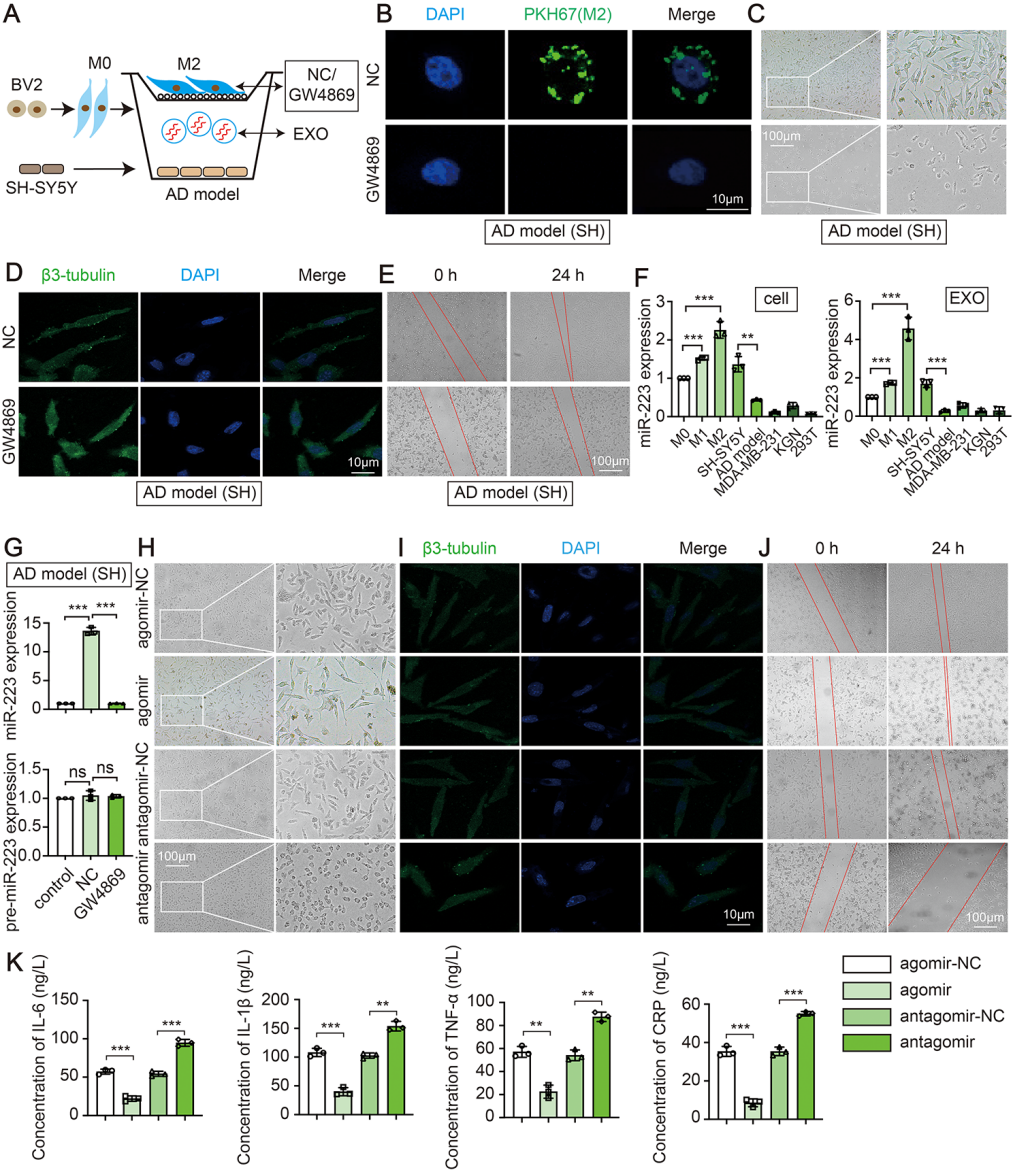
M2-like microglia derived exosomal miR-223 to improve AD cell model. (**A**) Schematic representation of coculture. (**B**) The internalization of PKH67-labeled EXO in the AD cell model of SH-SY5Y cell. Morphological characteristics (**C**), β3-tubulin immunofluorescence co-staining (**D**), wound healing assay (**E**) in the AD cell model of SH-SY5Y cell. (**F**) Cell and EXO sample of different cells were subjected to the measurement of miR-223. (**G**) The level of miR-223 and pre-miR-223 in AD cell model of SH-SY5Y cell. Morphological characteristics (**H**), β3-tubulin immunofluorescence co-staining (**I**), wound healing assay (**J**) and four inflammatory cytokines (**K**) in AD cell model of SH-SY5Y cell. (*N* = 3 independent experiments). Error bars represent means ± SEM. ns, no significance. **P < 0.01, ***P < 0.001 vs. control

Given the above evidence, we set out to investigate how anti-inflammatory microglia regulate nerve damage. A Transwell coculture system (Fig. 4A) was first established to test whether anti-inflammatory microglia derived EXO could be taken up by AD cell model. After 24 h, the AD cell model exhibited efficient uptake of EXO, while uptake disappeared with the inhibitor GW4869 (Fig. 4B and Figure S1B). Next, the cocultured system showed the improved cell morphology (Fig. 4C), increased synapses (Fig. 4D) and decreased scratch area (Fig. 4E) compared with the GW4869 group. Based on these results, anti-inflammatory microglia could ameliorate nerve damage through transporting EXO.

Our previous study identified a unique signature of miR-223 as potential protective for AD [19], thus, the levels of miR-223 in microglia and microglia-derived EXO (the characterization of EXO shown in Figures S1C-S1E) were assessed. Interestingly, miR-223 was enriched in anti-inflammatory microglia and anti-inflammatory microglia-derived EXO than that in AD cell model (SH), KGN (human ovarian granulosa tumor cell), MDA-MB-231 (breast cancer cell) and 293T cell (human embryonic kidney cell) (Fig. 4F). Moreover, miR-223, but not pre-miR-223, was significantly upregulated in AD cell model with anti-inflammatory microglia-derived EXO addition (Fig. 4G). The data indicate that miR-223 in AD cell model is derived from the transmission of microglia, rather than the pre-miRNA splicing of their own precursors. Then, the physiological impact of miR-223 were further assessed when overexpressing or inhibiting miR-223 in AD cell model. Overexpression of miR-223 significantly improved cell morphology (Fig. 4H), increased synaptic growth (Fig. 4I) and decreased scratch area (Fig. 4J) in AD cell model. In addition, the levels of IL-6, IL-1β, TNF-α and CRP were significantly downregulated in the miR-223-overexpressing pretreated AD cell model (Fig. 4K). While the inhibition of miR-223 had the opposite effect (Fig. 4H and K). Altogether, the results show that the elevation of miR-223 could associated with AD nerve damage repair.

### Involvement of YB-1 in microglia EXO sorting of miR-223

**Fig. 5:**
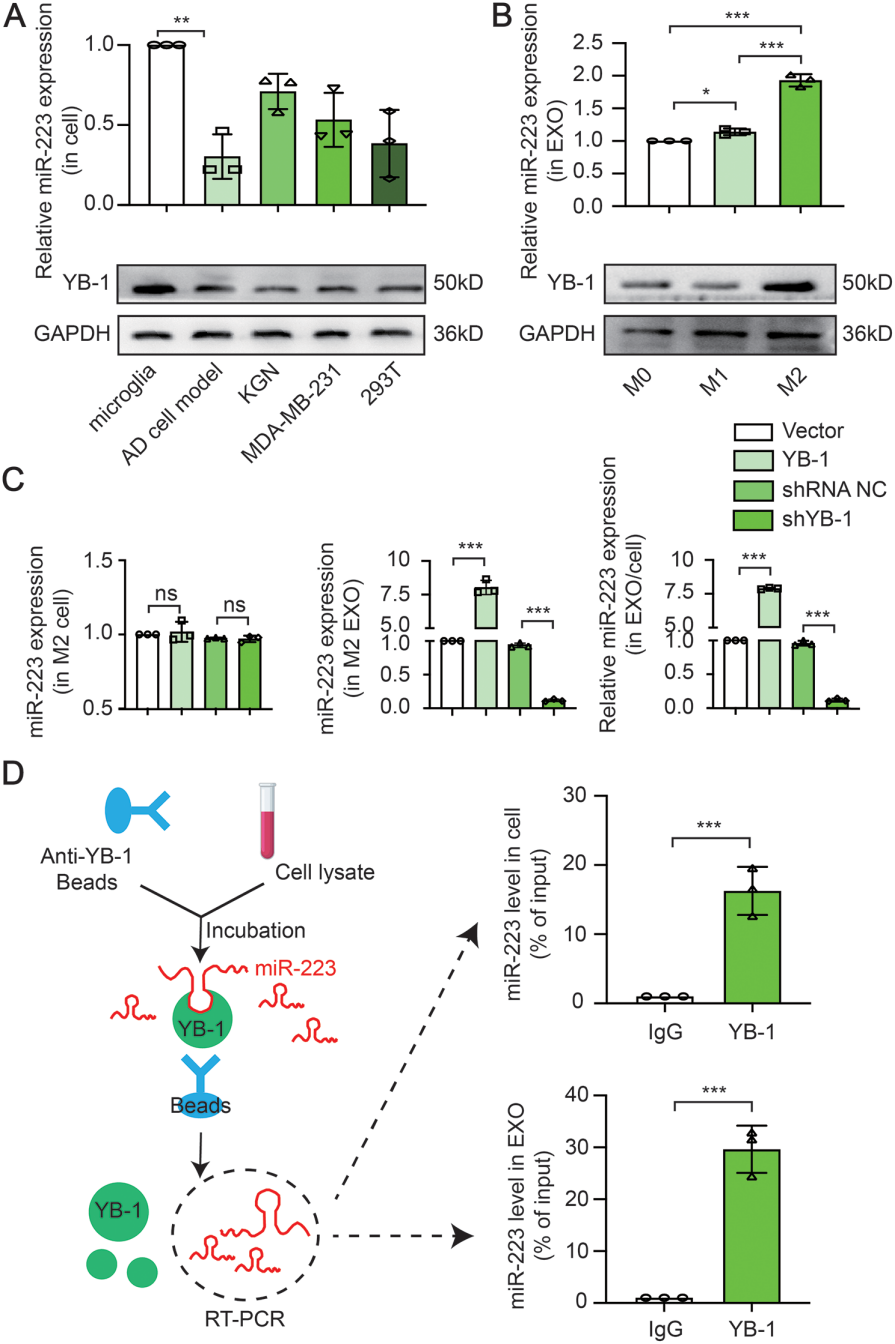
Involvement of YB-1 in microglia EXO sorting of miR-223. (**A**) The ratio of miR-223 in EXO/cell and YB-1 expression in different cells. (**B**) The ratio of miR-223 in EXO/cell and YB-1 expression in BV2. (**c**) The miR-223 level in cell and EXO sample in different groups. (**D**) RNA immunoprecipitation assay in M2 and EXO. (*N* = 3 independent experiments). Error bars represent means ± SEM. ns, no significance. *P < 0.05, **P < 0.01, ***P < 0.001 vs. control

Besides characterizing the function of miR-223 on AD cell model, we also sought to explore in further detail the mechanism by which miR-223 exerts its function. First, we found that the ratio of miR-223 in EXO/cell was reduced in AD cell model and was increased in anti-inflammatory microglia (Fig. 5A and B). Next, the mechanism of miR-223 specificity increased in EXO was further concerned. RNA binding protein (RBP) is recognized as one of the important mechanisms of EXO RNA/miRNA sorting. Interestingly, an increase of YB-1 (a kind of RBPs) accompanied by an increase in miR-223, was observed correspondingly (Fig. 5A and B). With the deepening of the scientific research, it has been reported that YB-1 is involved in the sorting of exosomal miR-223 in 293T cell [16]. To explore whether YB-1 binds to and is required for the sorting of miR-223, YB-1 was overexpressed or inhibited. Forced expression of YB-1 resulted in a significant upregulation of miR-223 in EXO, while deletion of YB-1 significantly decreased the level of miR-223 in EXO (Fig. 5C), which suggest that exosomal miR-223 is positively regulated by YB-1. However, the YB-1 has no effect on the expression level of miR-223 in the cells in anti-inflammatory microglia and AD cell model (Fig. 5C and S1F) and also PTEN level in AD cell model (Figure S1G), which further demonstrate that YB-1 is involved in the packaging of miR-223 into EXO rather than regulating regeneration or expression. To further define the interaction between miR-223 and YB-1, the RNA immunoprecipitation in anti-inflammatory microglia cell and EXO were performed. The results showed that miR-223 in cell is enriched in the RNA immunoprecipitation retrieved from the anti-YB-1 antibody group compared to that from the IgG group (Fig. 5D). Interestingly, the same results showed that YB-1 was efficiently immunoprecipitated from EXO extracts and was two times more efficient increased in EXO than that in cell (Fig. 5D). The data support that YB-1 directly interacts with miR-223 in cell and in EXO and is responsible for miR-223 EXO sorting.

### YB-1 mediated anti-inflammatory microglia exosomal miR-223 sorting attenuates nerve damage in vitro

The further study whether YB-1 is the active element responsible for the repairing impaired neurons properties mediated by exosomal miR-223 in AD cell model was demonstrated. Since microglia-derived EXO was able to transfer miR-223 to target cell, we anticipated that the EXO would present repairing impaired neurons properties. In a non-contact coculture system (Figs. 6-1A), overexpressed or inhibited YB-1 in anti-inflammatory microglia were co-cultured with AD cell model which overexpressed or inhibited miR-223. The overexpressed YB-1 in anti-inflammatory microglia lead to an increase of miR-223 in AD cell model, while knock down YB-1 accompanied by a decrease of miR-223 (Figs. 6-1B), which indicated that YB-1 could similarly transmit miR-223. miR-223 was reduced in miR-223 inhibited AD cell model when cocultured with YB-1 overexpressed anti-inflammatory microglia, which proved that mature miR-223, not pre-miR-223, is a regulator. The miR-223 was markedly increased in miR-223 overexpressed AD cell model when cocultured with YB-1 inhibited anti-inflammatory microglia (Figs. 6-1B), which proved that miR-223 was a direct regulator in AD. In addition, the same trend was found in AD cell model of PN (Figs. 6-2A-B).

**Fig. 6:**
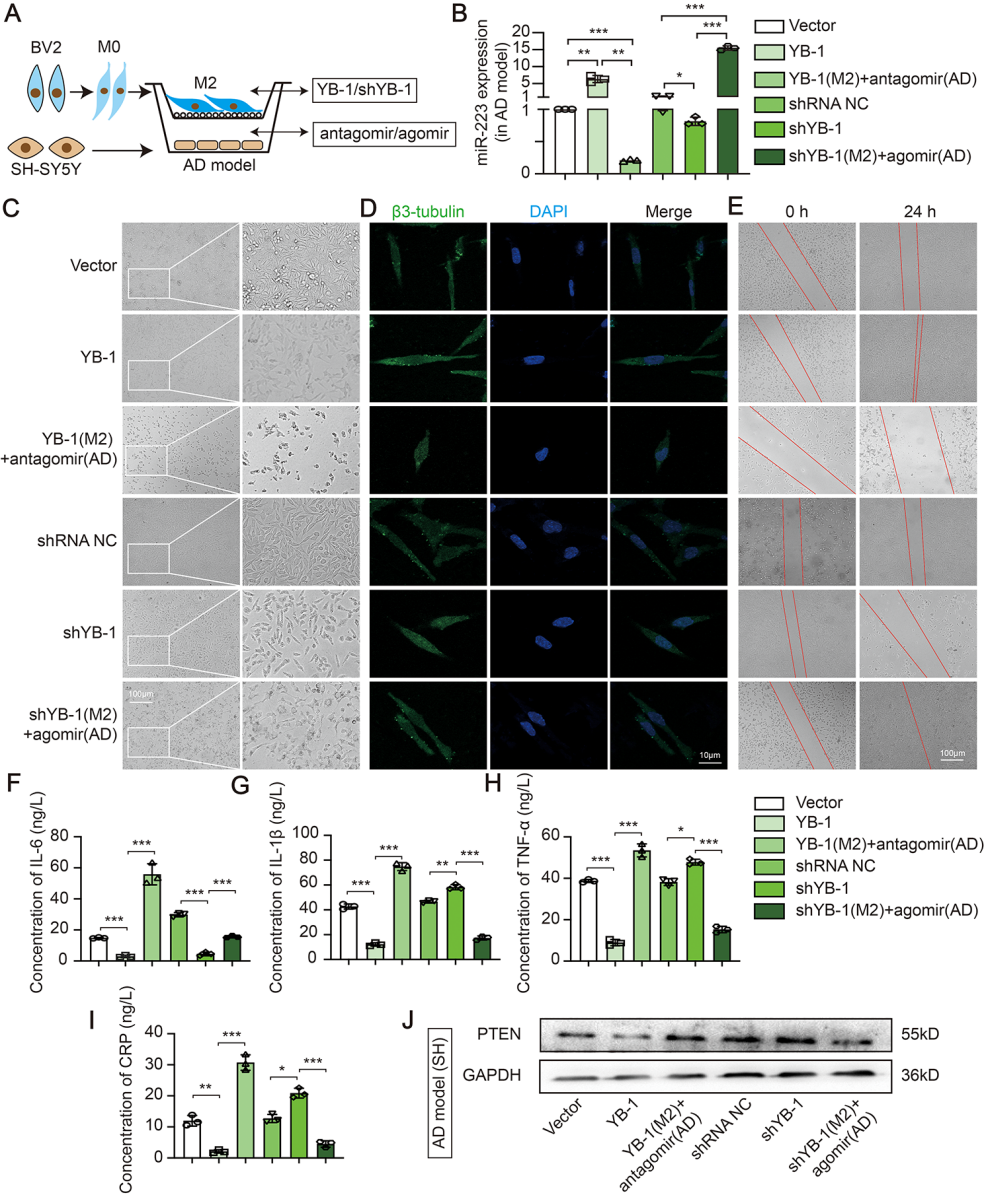
− 1. YB-1 mediated M2-like microglia exosomal miR-223 sorting attenuates nerve damage in *vitro*. (**A**) Schematic representation of coculture between M2 and SH-SY5Y cells. (**B**) The expression of miR-223 in AD cell model of SH-SY5Y cell. Morphological characteristics (**C**), β3-tubulin immunofluorescence co-staining (**D**), wound healing assay (**E**) and four inflammatory cytokines (**F**-**I**) in AD cell model. (**J**) The PTEN protein level in AD cell model. (*N* = 3 independent experiments). Error bars represent means ± SEM. *P < 0.05, **P < 0.01, ***P < 0.001 vs. control

**Fig. 6:**
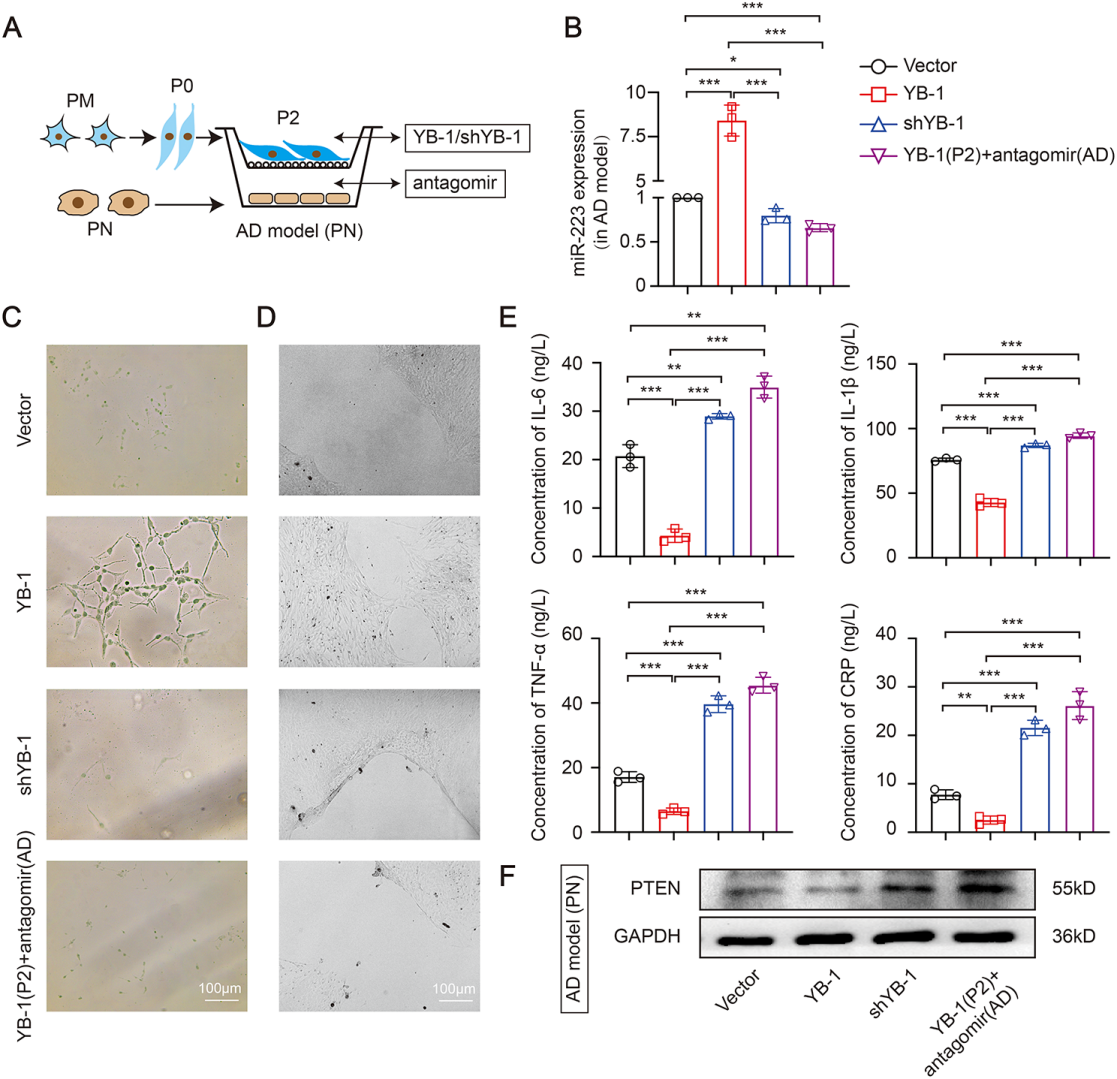
− 2. YB-1 mediated M2-like primary microglia exosomal miR-223 sorting attenuates nerve damage in *vitro*. (**A**) Schematic representation of coculture between PM and PN. (**B**) The expression of miR-223 in AD cell model. Morphological characteristics (**C**), wound healing assay (**D**) and four inflammatory cytokines (**E**) in AD cell model. (**F**) The PTEN protein level in AD cell model. (*N* = 3 independent experiments). Error bars represent means ± SEM. *P < 0.05, **P < 0.01, ***P < 0.001 vs. control

Subsequently, miR-223 overexpression in AD cell model reversed neuronal morphology damage caused by YB-1 depletion in anti-inflammatory microglia (Figs. 6-1C). The changes of synaptic growth (Figs. 6-1D) and ultimate wound healing (Figs. 6-1E) showed a same tendency compared with the results of morphology. Those observations emphasized that YB-1-packaged exosomal miR-223 could promote nerve repair. Then the similar effects of inflammatory cytokines were likewise observed (Figs. 6-1F-I), suggesting the release of pro-inflammatory factors were decreased by transporting of miR-223. In addition, the increased miR-223 level was accompanied by decreased PTEN express (Figs. 6-1J). Moreover, in AD cell model of PN, YB-1 overexpression in primary anti-inflammatory microglia group similarly improved cell morphology (Figs. 6-2C), decreased scratch area (Figs. 6-2D), and downregulated inflammation (Figs. 6-2E). Also, the increased miR-223 level was accompanied by decreased PETN express (Figs. 6-2F). As shown in Figs. 6 − 1 and Figs. 6 − 2, miR-223 is a direct regulator in improving nerve damage. Collectively, these findings delineate a process whereby YB-1 is responsible for sorting miR-223 into microglia EXO and could attenuate nerve damage.

### EXO derived from anti-inflammatory microglia attenuate neuroinflammation and cognitive impairments in vivo

**Fig. 7:**
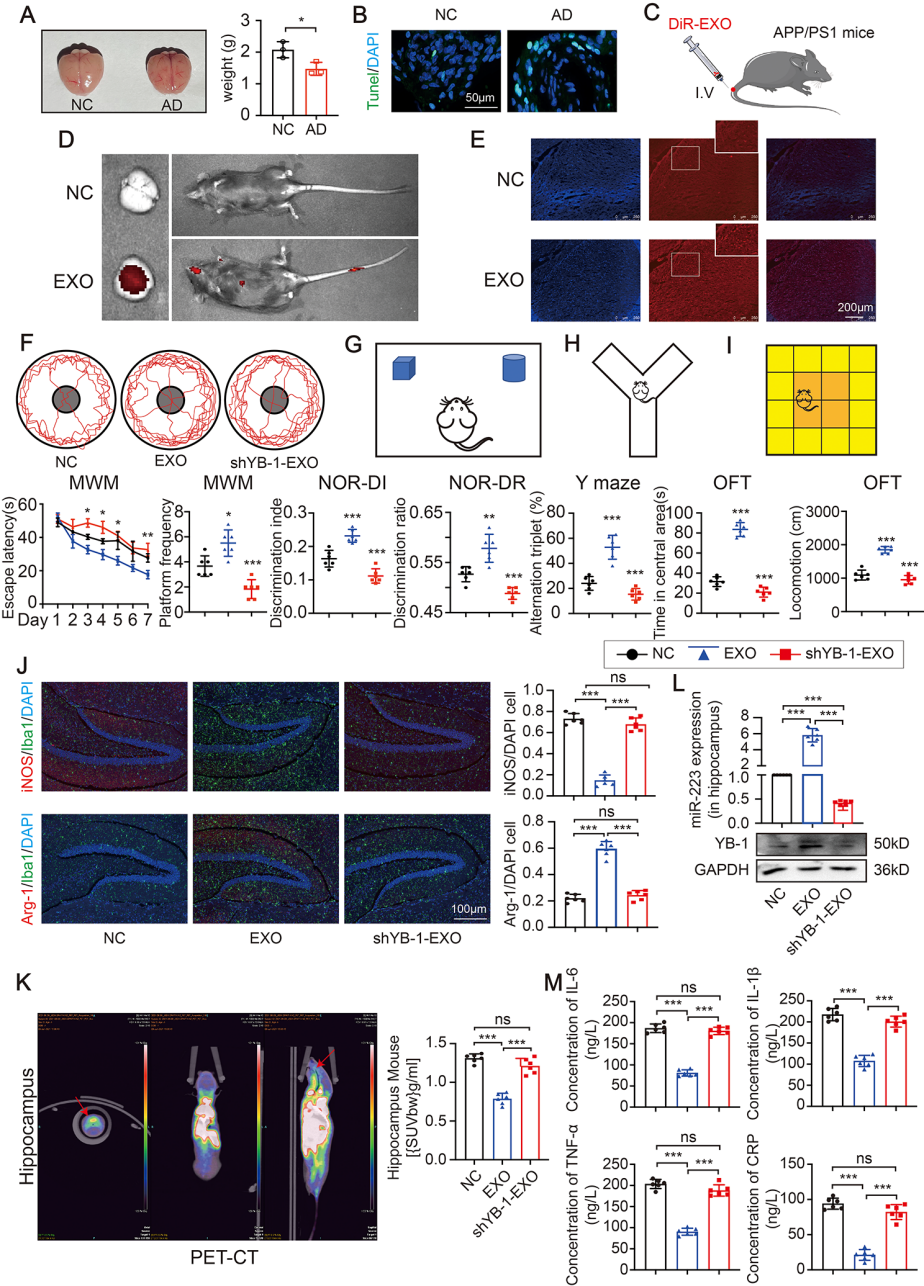
EXO derived from M2-like microglia attenuate neuroinflammation and cognitive impairment in *vivo*. (**A**) The brain image and the weight of the brain. (**B**) The Tunel analysis in hippocampus. (**C**) Schematic representation of intravenous EXO. (**D**) The representative IVIS images of mice. (**E**) Representative fluorescence images of the DiR-labeled EXO in brain tissue. (**F**) Escape latency and platform frequency of the Morris water maze test. (**G**) Discrimination index and discrimination ratio of the novel object recognition task. (**H**) Alternation triplet of Y maze tests. (**I**) Time spent in central area and total locomotion of open field test. (**J**)The microglia markers in the hippocampus of brain tissue. (**K**) The representative PET-CT images and accumulation of [18 F] DPA-714 in hippocampus. (**L**) The miR-223 and YB-1 expression in hippocampus. (**M**) The four inflammatory cytokines in hippocampus. (*N* = 6 independent animals). Error bars represent means ± SEM. ns, no significance. *P < 0.05, **P < 0.01, ***P < 0.001 vs. control

**Fig. 8:**
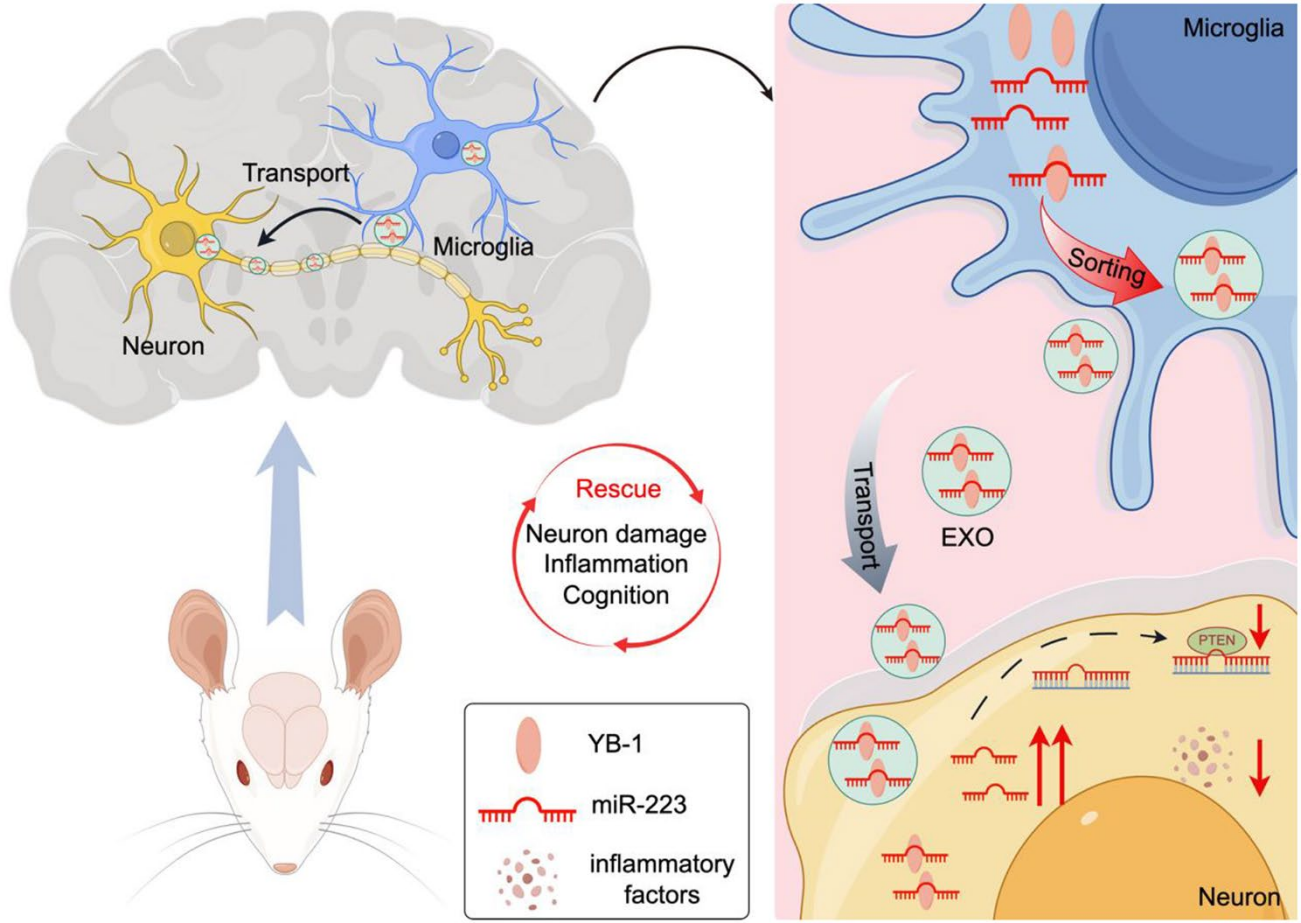
The proposed model illustrating the property of microglia-derived EXO in the AD context

First, compared with the normal mice, the APP/PS1 mice (a kind of transgenic mice having cerebral Aβ plaques) had a shrunken brain (Fig. 7A-B). To test the hypothesis that EXO could ameliorate brain damage in vivo, we intravenously injected DiR-labeled anti-inflammatory microglia-derived EXO and examined the brain after infusion (Fig. 7C). DiR hotspots in DiR-EXO infused animals were localized predominantly in brain and liver (Fig. 7D). Importantly, the DiR-EXO was also able to reach the brain tissue (Fig. 7E). In addition, the behavioral testing including MWM, NOR, Y maze tests, and OFT indicated that APP/PS1 mice displayed the cognitive deficits when compared with the normal mouse (Figure S2). Also, treatment with DiR-EXO dramatically rescues the spatial learning and memory deficits of AD mice (Fig. 7F-I). Next, the proportion of CD68 was reduced and the proportion of CD206 was increased in the EXO treated group, which indicated that treatment with EXO could reduce the neuroinflammation (Fig. 7J). A reduced mean [18 F] DAP-714 uptake in the hippocampus was observed. The result indicated that microglial activation and neuroinflammation were improved in EXO treatment group, while inhibition of YB-1 was associated with the worsening inflammation (Fig. 7K). Moreover, the levels of miR-223 and YB-1 were increased in EXO group, while reduced in shYB-1-EXO group (Fig. 7L). And consistent with the above results, the corresponding changes in inflammatory cytokines in hippocampus was observed (Fig. 7M). In summary, these findings support that YB-1-mediated microglial EXO loading of miR-223 directly reduce inflammation in brain, improve cognition and highlight the capacity of anti-inflammatory microglia-derived EXO for functional restoration in vivo (Fig. 8).

### Correlation of exosomal miR-223/YB-1 axis and cognitive performance in human subjects

**Fig. 9:**
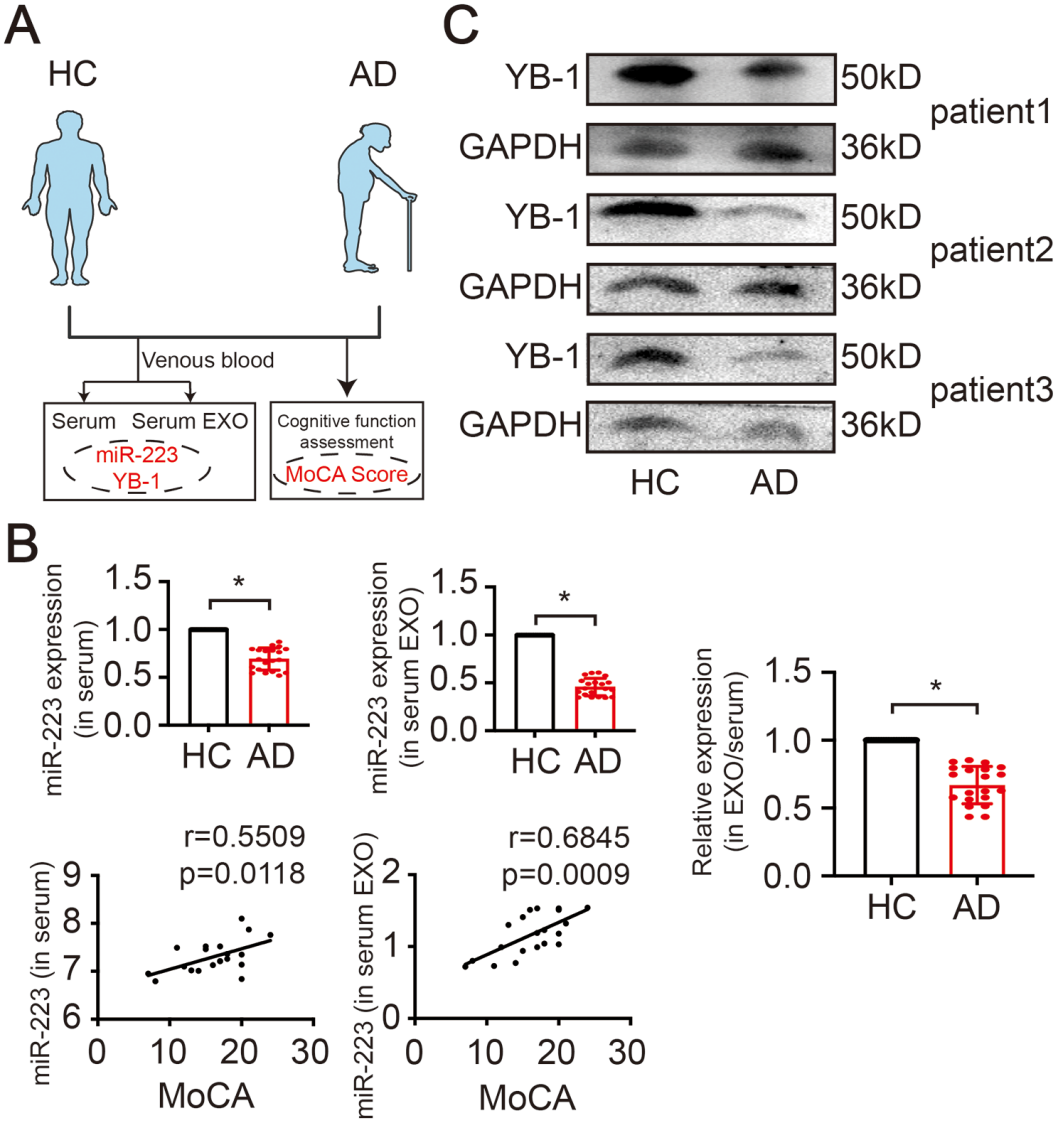
Correlation of exosomal miR-223/YB-1 axis and MoCA in human subjects. (**A**) Schematic representation of enrolled participants. (**B**) Serum and serum EXO sample of AD patients and healthy controls were subjected to the measurement of miR-223, and the correlation between miR-223 and MoCA. (N=20 independent individuals). (**C**) The YB-1 expression in serum. (*N*=3 independent experiments). Error bars represent means?SEM. *P < 0.05, **P < 0.01, ***P < 0.001 vs control

Next, we examined the clinical significance of miR-223 and YB-1 (Fig. 9A). The level of miR-223 was decreased in both AD patients’ serum and serum-derived EXO. In parallel, the relative expression level of miR-223 in the ratio of EXO/serum present a decrease in AD (Fig. 9B). And all participants were subjected to cognitive evaluation with MoCA tests, and their cognitive status was correlated with serum miR-223 and exosomal miR-223 (Fig. 9B). Furthermore, the expression of YB-1 in serum was decreased in AD patient (Fig. 9C). Taken together, these data suggest that miR-223 and YB-1 expression profile are altered in AD and the level of miR-223 was correlated with cognitive status.

## Discussion

An increased appreciation that neuroinflammation is a major driving force in CNS pathologies has led to an explosion of interest in microglia in conditions including AD [23]. In this study, we first demonstrated that AD model could lead to the polarization of M1-like pro-inflammatory microglia, which in turn promotes the progression of neuroinflammation. On the other hand, we functionally confirmed that M2-like anti-inflammatory microglia derived EXO attenuate damaged neuron and reverse cognition in *vitro* and in *vivo*. Mechanistically, YB-1 directly interacted with miR-223 both in cell and EXO, and participated in microglia exosomal miR-223 loading. One unique attribute of our study is that we demonstrate that M2-like anti-inflammatory microglia-derived EXO-miR-223 may be a potential therapy in AD.

As one of the most abundant immune cell, microglia play a crucial role in the AD microenvironment [24]. The release of pro-inflammatory molecules can lead to synaptic dysfunction, neuronal death and inhibition of neurogenesis [25]. In our study, we confirmed that AD model could promote the polarization of M1-like microglia, and M1-like microglia could further promote the progression of AD. Correspondingly, Simeoli et al. found that damaged neurons could increase M1 polarization and decrease M2 polarization [26]. On the other hand, M2 are associated with the attenuation of inflammation [27], which is manifested in strategies harnessing microglia are effective in clearing parenchymal and vascular Aβ in the brain and attenuating the progression of inflammation in mouse models of AD [28]. In accordance with our findings, EXO secreted from M2-like microglia could be efficiently taken up and alleviated inflammation in AD in *vitro* and in *vivo*. Collectively, these findings suggest that a complex interplay between the AD cell model and M1 pro-inflammatory microglia, and M2 anti-inflammatory microglia could repair neuron damage.

Cell respond to inflammatory states by releasing EXO containing highly specific cargos [12, 29]. Exosomal-associated miRNAs can modulate the expression of target genes and affect neurons in recipient cell after internalization. Our previous studies have shown that exosomal miR-223 could downregulate inflammation in AD [9, 30]. Here, we found M2-derived exosomal miR-223 could enhance regulatory role by vesicle mediated internalization in *vitro* and in *vivo*. Importantly, adoptive transfer of EXO enriched with miR-223 could reduce neuron damage and neuroinflammation. Moreover, we also establish the cross-talk between nerve cell and microglia and subsequently repaired neuroinflammation, which may provide a novel molecular target for the AD therapy.

Cells communicate with each other via the transfer exosomal cargo, and the miRNA content can play a critical role in cell-cell communication [6]. Recently, studies have found that YB-1 is required to sort mRNAs and miRNAs into EXO [16, 19]. In this study, we demonstrated that YB-1 enriched exosomal miR-223 in M2, and transmitted miR-223 to AD cell model. These data clearly showed the mechanistic link through which the YB-1/exosomal miR-223 axis mediated cellular communication between nerve cells. In subsequent studies, we provided evidence that YB-1 could interact with miR-223 in both cell and EXO. It has considered that YB-1 could recognize and bind specific motifs, such as ACCAGCCU, CAGUGAGC and UAAUCCCA [31, 32], and control miRNAs sorting into EXO. Whether YB-1 contains RNA-binding domains to interact with miR-223 has not yet been studied and future investigations will be needed. And we compared the relevant sequences and found no coincidence, suggesting that the sorting of miR-223 by YB-1 may be accomplished by other special mechanisms. Our data support the findings that YB-1 were capable of sorting miR-223 into EXO. However, the specific sorting mechanism of the interaction between YB-1 and miR-223 remains to be further studied.

It has been reported that EXO could cross the blood-brain barrier and deliver cargo to the brain [7]. Our study demonstrated that the EXO injected via tail vein could reach the liver and brain, although dose reaching the brain was generally low when compared to peripheral organs. Moreover, after injection of M2-EXO, the level of inflammatory factors was significantly downregulated, and the miR-223 expression was significantly increased. Together, M2-derived exosomal miR-223 could alleviate neuroinflammation and repair cognitive damage in AD. These findings are of interest to basic understanding of EXO to practical applications in delivery to central nervous system.

## Conclusions

In summary, we demonstrated that exosomal miR-223 mediated the cross-talk between AD cell model and microglia, that the protect properties of microglia-derived EXO due in part to the delivery of miR-223 to AD model in *vivo* and in *vitro*. Mechanistically, we identified an unsuspected role for YB-1 in determining EXO cargo specificity, like sorting miR-223 into EXO. This work expands our understanding of the regulation of cellular trafficking and EXO biogenesis in AD, and thus provides important and novel insights into the potential application of EXO as therapeutic.

### Electronic supplementary material

Below is the link to the electronic supplementary material.

Supplementary Material 1

Supplementary Material 2

Supplementary Material 3

## Data Availability

Data contained within the article or supplementary material are available from the corresponding author upon reasonable request.

## Abbreviations

AD: Alzheimer’s Diseases
EXO: Exosome
RIP: RNA-Binding Protein immunoprecipitation
MSC: Mesenchymal Stem Cell
MVB: Multivesicular Body
RBPs: RNA Binding Proteins
PN: Primary Neuron
PM: Primary Microglia
IRW: Inveon Research Workplace
ROI: Region of Interest
