# Supplementary figures and images for: Microglia-derived exosomes selective sorted by YB-1 alleviate nerve damage and cognitive outcome in Alzheimer’s disease
Source: J Transl Med. 2024 May 16;22:466. doi: 10.1186/s12967-024-05256-x (PMC11100039; doi:10.1186/s12967-024-05256-x)

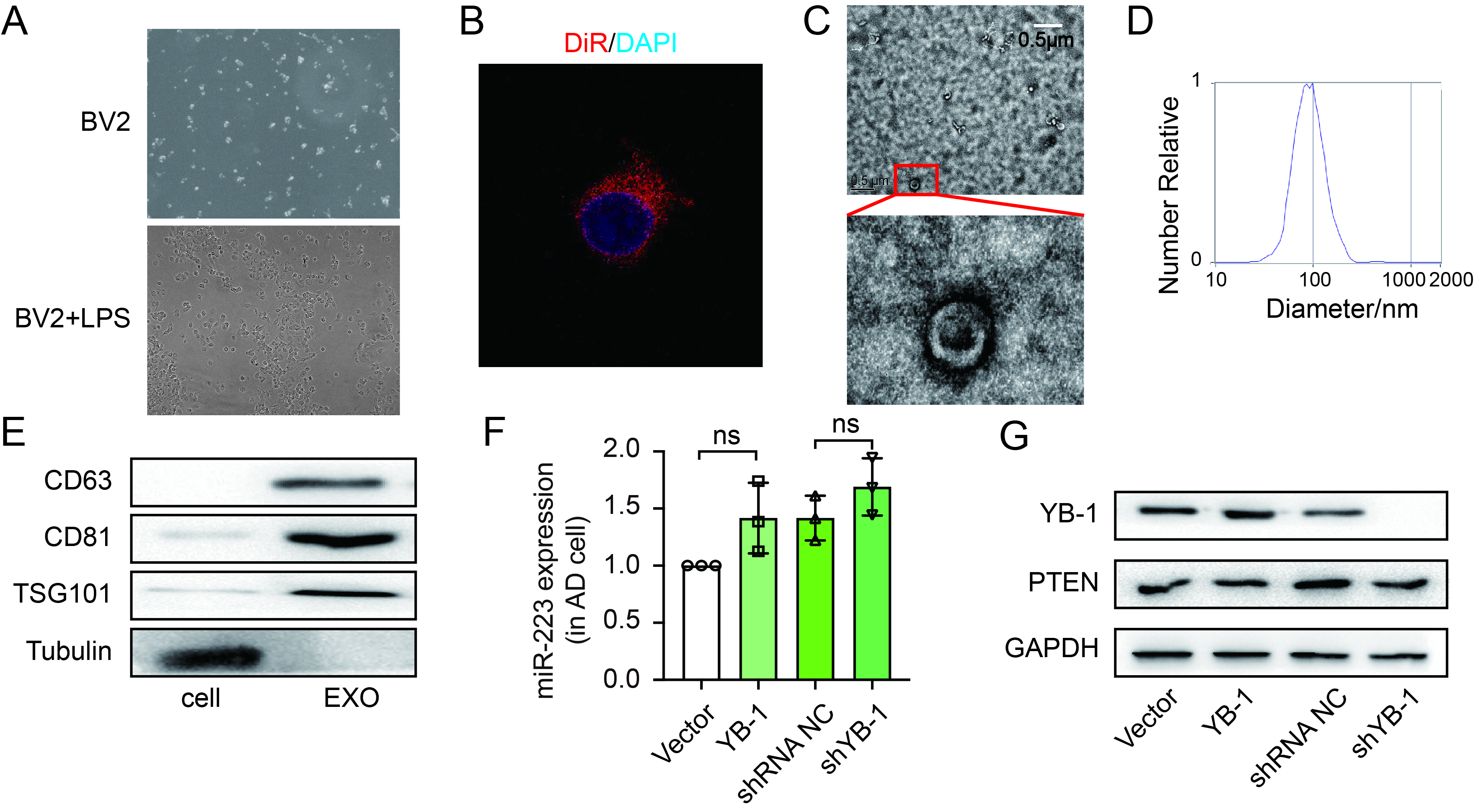

Supplement: Supplementary file 1 — Supplementary Material 1 [file 12967_2024_5256_MOESM1_ESM.tif]

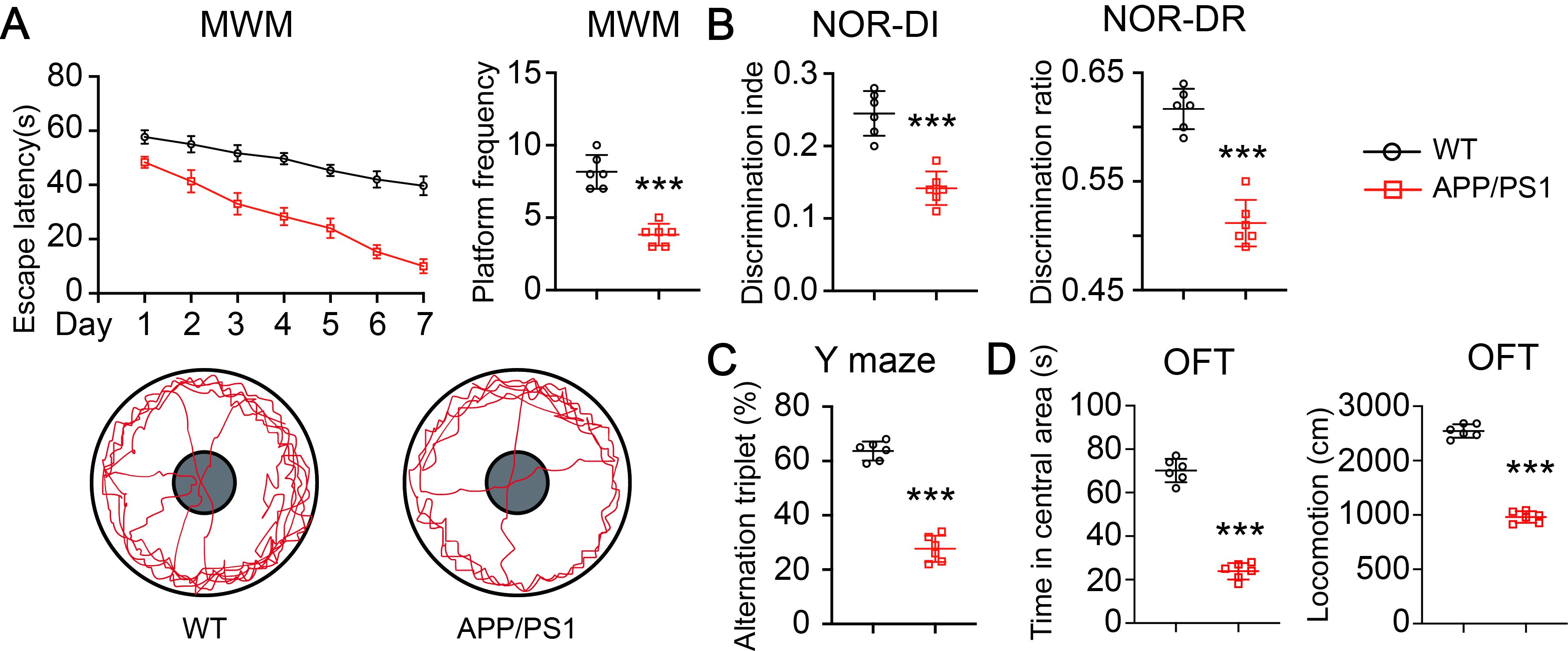

Supplement: Supplementary file 2 — Supplementary Material 2 [file 12967_2024_5256_MOESM2_ESM.tif]
