# Supplementary material for: Microglia-derived exosomes selective sorted by YB-1 alleviate nerve damage and cognitive outcome in Alzheimer’s disease
Source: J Transl Med. 2024 May 16;22:466. doi: 10.1186/s12967-024-05256-x (PMC11100039; doi:10.1186/s12967-024-05256-x)
Supplement: Supplementary file 3 — Supplementary Material 3 [file 12967_2024_5256_MOESM3_ESM.docx]

Dear Editors and Reviewers:

Thank you for the reviews’ comments concerning our manuscript entitled “Microglia-derived exosomes selective sorted by YB-1 alleviate nerve damage and cognitive outcome in Alzheimer’s disease” (ID: JTRM-D-23-08744). We have studied comments carefully and have made correction which we hope met with approval. The main corrections in the paper and the responds to the reviewer’s comments are as following:

Reviewer #1: Excellent in-vitro and in-vivo work to elucidate the role of M1 and M2 microglia derived exosomes, and their subsequent effect on neuronal injury.

Reply: Special thanks to you for your good comments.

Needs English and grammar corrections.
Reply: Thank you for your recommendation. After receiving your valuable reply, we sought help from English proofreading company AJE to improve the quality of our manuscript. Moreover, we carefully proof-read the manuscript to minimize grammatical and bibliographical errors. We hope it will be better this time.

Reviewer #2: The conclusions of this paper are novel and interesting enough for the audience working in the field. However, a deep, substantial review is needed to increase the quality of the manuscript.

Reply: Special thanks to you for your good comments.

1. In an effort to increase rigor and standardization in the study of EVs, the International Society for Extracellular Vesicles (ISEV) established in 2018 the Minimal Information for Studies of Extracellular Vesicles 2018 (MISEV2018, C. Théry and K.W.Witwer, et al., 2018, PMID: 30637094) guidelines. The characterization of EVs reported in this work does not comply to it.

Reply: Thank you for your helpful comments. We have studied in detail the properties of EXO in this article (PMID:30637094), which mentioned that EVs characterization include transmembrane or GPI-anchored proteins associated to plasma membrane and/or endosomes and cytosolic proteins recovered in EVs. The transmembrane or GPI-anchored proteins including CD63 and CD81 which already mentioned in our manuscript. The cytosolic proteins TSG101 were validated, and we confirmed that the EXO obtained in our manuscript met the international standards as described in above article. The results have been added in revised manuscript in Figure S1C.

2. Data that are not shown or inferred from literature must be avoided. Please show what it is stated in the results section, or eliminate sentences referring to unshown data. For example, 'Indeed, our previous studies found that miR-223 movement from MSC to AD cell model through EXO could protect against nerve injury'; 'After differentiation, round and floating microglia cell became adherent flattened cell (data not shown)'.

Reply: Thank you for your valuable suggestion. The methods and morphological changes of microglial polarization are classically recognized. In our experiments, microglia changed from round and floating cells to adherent flattened cell after induced differentiation. And, we have supplemented the morphology of the BV2 microglia cells before and after differentiation in Figure S1A in the revised manuscript.

3. The n for all the experiments, not just for the behavioral data. It should be reported in the relative figure legends.

Reply: Thank you for your suggestion. In our manuscript, in vitro experiment was repeated 3 times (n=3 independent experiments), and in vivo experiments were performed with 6 mice per group (n=6 independent animals). The serum and serum EXO sample of AD patients and healthy controls were 20 sample per group for the PCR (n=20 independent individuals). And we mixed EXO derived from three patient serum as one sample for the Western Blot Assay (n=3 independent experiments). Statistical analyses were performed on the experiments including behavioral experiments and marked in the Figures. We have followed the reviewer’s suggestion and added these in the corresponding figure legends and Methods.

4. 'Morris water maze, novel object recognition and Y maze tests results indicated that DiR-EXO dramatically rescues the spatial learning and memory deficits of AD mice (Figure 7F-H)'. The conclusion is not sufficiently supported by the data and requires additional experimentation. Furthermore, in vivo tests, the normal mouse and AD model group should be included as the negative and positive control, respectively.

Reply: Thank you for your good suggestion. In our manuscript, the Morris water maze test evaluates spatial learning and memory, novel object recognition test evaluates recognition memory, and Y maze test evaluates short-term memory. In addition, the open field tests (OFT) including time spent in center area and total locomotion were added to further support our conclusion in Figure 7I in revised manuscript. Moreover, we have supplemented the behavioral experiments in the Methods section. Followed the reviewer’s suggestion, we have also added the results of cognitive differences between the APP/PS1 mice and normal mouse in the Figure S2.

5. In the schematic illustration, it is hard to understand the goal or concept of this study. Please improve the quality of illustration for readers. Also, I recommend you to unify the fonts of the schematic illustration, and to refine the overall composition and picture to look better.

Reply: Thank you for your suggestion. Accordingly, we modified the schematic accordingly and refine the figure in the schematic illustration in Figure 9. In particular, the research concept of this manuscript, which YB-1-mediated microglial exosomal sorting of miR-223 participates in alleviating nerve damage, is emphasized. Also, we unify the fonts and upload higher quality figures than before. The schematic illustration has been modified accordingly in the revised manuscript.

6. All the data have poor resolution. Please improve the resolution of the "all" the data including graphs and figures. In most graphs, the numerical value is hard to read. In all the figures using confocal microscopy, the resolution is too low to figure out the result.

Reply: Thank you for your suggestion. It is possible that the quality of figures was reduced due to their conversion to PDF format. We have uploaded each image separately, along with improve the figures resolution to 600dpi, and we also inserted the original figures into the WORD format to look better. And we also changed some pictures to make it easier to read.

7.The description in Figure 9 is confusing. Is the exosome in the nucleus?

Reply: I would like to express my appreciation to the valuable comments of the reviewer. Due to our poor drawing technique, we mistakenly drew EXO inside the nucleus. According to our study, miR-223 is sorted into EXO in the cytoplasm. We have modified the schematic illustration in the revised manuscript.

8. The wording is not rigorous or standardized. Aβ1-40 was added to SH-SY5Y cells for AD cell model. But it cannot be described as an AD model.

Reply: Thank you for your good suggestion. We have changed the “AD model” in this manuscript into “AD cell model” or “AD mouse model” in the revised article in red. And the “AD cell model” and “AD mouse model” are collectively called “AD model”.

9.Some experimental details are missing. For example, how many EVs per ml of culture or per cells were added? How many EVs were injected in animal experiments? More details on the EV-producing cultures and volumes of conditioned media required for EV isolation should be provided. How were behavioral experiments conducted? The author needed to provide specific methods and details.

Reply: Thank you for your valuable suggestion. In the in vitro experiment, it was a direct co-culture system of cell and cell, so there was no extra addition of EXO. After inducing the phenotypes, the medium of BV2 cells was replaced with EV-free medium for 48 hours. Then, the EXO pellets were collected from the supernatant of M2-like microglia. The EXO isolated from the supernatant of about 1×10^7^ cells (two 25cm^2^ petri dish), was used for one injection. The total volume of EXO for per mouse was approximately 2*10^10^. All of the above have been added in the Method part in revised manuscript. In addition, the detail of behavioral experiments including Morris water maze (MWM), novel object recognition (NOR) and Y maze tests also have been added in the corresponding part in red.

10. The grammar is very poor, and should be deeply reviewed by someone who is more proficient in English. For example, the abstract and the introduction are almost the same, and they do not clearly explain the necessity and content of the study. The full name of the first abbreviation shall be written clearly. For example, page 4 line89 MVB.

Reply: Thank you for your suggestion and we have sought the help of the professional English company AJE for improving the quality of article. We also read the article carefully and revised it correspondingly for better understanding. Moreover, we removed some content with little relevance to the topic and made a concise description for better highlighting the experimental content and necessity in the Introduction part. The abbreviation referred has been corrected in red, and we checked and revised all the abbreviations in the manuscript.

11. PKH-67 is one of the worst dyes to stain EVs, given that the dye itself when ultracentrifuged can form contaminating nanoparticles of the same size of EVs (as shown by P.P. Dominkus, et al., 2018, PMID: 29551275). Thus, it is impossible to distinguish between contaminating dye-only particles and genuine EVs in Fig. 2A and 3D. The authors should use a different dye (for instance, CellMask from ThermoFisher) or should load the stained EVs on a preparative sucrose-gradient cushion to eliminate PKH-nanoparticles, as shown by Dominkus et al., 2018.

Reply: Thank you for your valuable suggestion. The Fig.2A and 3D showed the staining of β3 tubulin, which is a marker of neuron. As suggested by the reviewer and summarized in the journal published in 2018 by P.P. Dominkus et al., there may be some deficiencies in the staining of PKH67 exosomes. Due to the limited revised time, the purchase and experimental verification of new dyes were not allowed. But we performed the EXO tracking in vitro using the available DiR dye, and got the same conclusion. And we have added the uptake of DiR-labeled EXO in vitro in the Fig S1B in the revised manuscript. Moreover, some articles are labeling EXO with PKH67 and/or DiR dyes (as shown by J. Hu. et al., 2022, PMID:35883151; V.V. Nguyen. et al., 2022, PMID:36382606; X. Gao. et al., 2023, PMID:37535897). Thanks to the reviewer for this suggestion, we will try to use other better dyes in the future work in order to obtain better conclusion. Thanks again for the meaningful comments.

12. Blots should report where the protein molecular-weight size markers are and not the molecular weight of the single proteins. Ideally, the area cut from the blot should be big enough to contain two molecular-weight references

Reply: We sincerely thank the reviewer for pointing out this problem, and we have been added the molecular weight of the proteins in the revised version accordingly and marked in red. Due to our lack of experience, individual proteins were cut in the WB experiments to save antibody cost, so that only the original picture of a single blot could be provided. But we have provided the original blots of all blots with molecular weight, and we also marked the marker location based on the recording of the original blots. In the future experiments, we must use the whole membrane for Western Blotting in order to obtain a more representative conclusion.

13. The author's rescue experiment design is unreasonable. 'miR-223 was reduced in miR-223 inhibited AD cell model when cocultured with YB-1 overexpressed anti-inflammatory microglia, but markedly increased in miR-223 overexpressed AD cell model (SH) when cocultured with YB-1 inhibited anti-inflammatory microglia (Figure 6-1B)'. The miR-223 was reduced in miR-223 inhibited AD cell model, but markedly increased in miR-223 overexpressed AD cell model. That's just the way that goes.

Reply: Thanks so much for your valuable suggestions. With the question raised by the reviewer, we repeated the experiment again and still hold the same results. Our results proved that, in our rescue experiment design, overexpressed or inhibited in microglia demonstrated that YB1 was an upstream factor of miR-223 and acted as a sorting miR-223. As indicated in the comments, in the group “shYB-1 (M2) + agomir (AD cell)” proved that miR-223 is a direct regulator of improvement in nerve damage function. As shown in Figure4G, YB-1 delivered the mature miR-223, not pre-miR-223, to the AD cell model. But the antagomir was competitively binds to miR-223, including its endogenous miR-223 and the transmitted exogenous miR-223, and lost its functions. However, in the group “YB-1 (M2) + antagomir (AD cell)”, inhibition of miR-223 in AD cell model resulted in a decrease in both endogenous and exogenous miR-223 levels in AD cell model, and its function was affected accordingly. This result also proved that miR-223, not pre-miR-223, is a direct regulator in AD. For better understanding, we have made some modifications to the presentation in revised manuscript.

14. 'The changes of synaptic growth (Figure 2C) and ultimate wound healing (Figure 2D) showed a same tendency compared with the results of morphology'. There is a lack of statistical result graphs, and these conclusions cannot be drawn. 'First, compared with the normal mice, the APP/PS1 mice (a kind of transgenic mice having cerebral Aβ plaques) had a shrunken brain (Figure 7A)'. This part also lacks statistical results.

Reply: Thank you for pointing out this. We have followed the reviewer’s suggestion and have added the statistical results in the figure 2C, 2D and 7A correspondingly.

We tried our best to improve the manuscript and made some changes in the manuscript. We sincerely appreciate the Editor’s and Reviewers’ hard work and hope that the corrections will be met with approval. If you have any questions, please feel free to contact us.

Once again, thank you very much for your comments and suggestions.

Yours sincerely,

Xiaolan Zhu
